# Supplementary material for: Semantic priming supports infants’ ability to learn names of unseen objects
Source: PLoS One. 2021 Jan 7;16(1):e0244968. doi: 10.1371/journal.pone.0244968 (PMC7790528; doi:10.1371/journal.pone.0244968)
Supplement: S1 Appendix — (PDF) [file pone.0244968.s001.pdf]

MacArthur Short Form
Vocabulary Checklist: Level I

Copyright 1993 All Rights Reserved*
*For information/copies, contact the Developmental
Psychology Lab, San Diego State University, San Diego, CA 92182

Make no stray marks.
Erase any changes cleanly.

Improper Marks ⊘⊗◒⊙ **Proper Mark** ●

USE NO. 2 PENCIL ONLY

Child's Name ______________________ **Sex** __________

Birthdate ______________________ **Today's Date** ______________________

VOCABULARY CHECKLIST

For words your child understands but does not yet say, mark the first column (understands). For words that your child not only understands but also says, mark the second column (understands and says). If your child uses a different pronunciation of a word, mark it anyway.

	UNDERSTANDS	UNDERSTANDS AND SAYS		UNDERSTANDS	UNDERSTANDS AND SAYS		UNDERSTANDS	UNDERSTANDS AND SAYS
choo choo	○	○	chair	○	○	wait	○	○
meow	○	○	couch	○	○	break	○	○
ouch	○	○	kitchen	○	○	feed	○	○
uh oh	○	○	table	○	○	finish	○	○
bird	○	○	television	○	○	help	○	○
dog	○	○	blanket	○	○	jump	○	○
duck	○	○	bottle	○	○	kick	○	○
kitty	○	○	cup	○	○	kiss	○	○
lion	○	○	dish	○	○	push	○	○
mouse	○	○	lamp	○	○	sing	○	○
car	○	○	radio	○	○	smile	○	○
stroller	○	○	spoon	○	○	night	○	○
ball	○	○	flower	○	○	today	○	○
book	○	○	home	○	○	all gone	○	○
doll	○	○	moon	○	○	big	○	○
bread	○	○	outside	○	○	broken	○	○
candy	○	○	plant	○	○	dark	○	○
cereal	○	○	rain	○	○	fast	○	○
cookie	○	○	rock	○	○	hurt	○	○
juice	○	○	water	○	○	pretty	○	○
toast	○	○	babysitter	○	○	soft	○	○
hat	○	○	girl	○	○	I	○	○
pants	○	○	grandma	○	○	me	○	○
shoe	○	○	mommy	○	○	how	○	○
sock	○	○	bath	○	○	who	○	○
eye	○	○	don't	○	○	away	○	○
head	○	○	hi	○	○	out	○	○
leg	○	○	night night	○	○	other	○	○
nose	○	○	patty cake	○	○	some	○	○
tooth	○	○	please	○	○			

cat	O	O	orange	O	O
horse	O	O	bus	O	O
apple	O	O	truck	O	O
banana	O	O	jacket	O	O
