# Supplementary material for: Semantic priming supports infants’ ability to learn names of unseen objects
Source: PLoS One. 2021 Jan 7;16(1):e0244968. doi: 10.1371/journal.pone.0244968 (PMC7790528; doi:10.1371/journal.pone.0244968)
Supplement: S2 Appendix — (PDF) [file pone.0244968.s002.pdf]

Questionnaire

Our research receives funding from the government. The agencies which are funding us are interested in the diversity of our participants. Your answers to these questions will remain confidential and will not in any way be connected with your child's study results.

Your Child

Is your child a male or female?

☐ Male

☐ Female

What race do you consider your child to be? Please check all that apply.

☐ American Indian or Alaskan Native

☐ Asian

☐ Native Hawaiian or Other Pacific Islander

☐ Black or African American

☐ White

Do you consider yourself to be Hispanic or Latino?

☐ Hispanic or Latino

☐ Not Hispanic or Latino

Is there anything further you would like to add about your child's race or ethnicity heritage?

____

____

PARENT 1

☐ Male ☐ Female

EDUCATION

1. Check the highest educational degree you have attained.

☐ None- what is the highest grade completed?

____

☐ High School Diploma

☐ Associate Degree

☐ Vocational Degree

☐ Bachelor's Degree

☐ Master's Degree

☐ Ph.D, J.D., or M.D.

OCCUPATION

1. What is your occupation?

____

2. Are you currently working?

☐ Full-time ☐ On leave

☐ Part-time ☐ Not working

PARENT 2

☐ Male ☐ Female

EDUCATION

1. Check the highest educational degree you have attained.

☐ None- what is the highest grade completed?

____

☐ High School Diploma

☐ Associate Degree

☐ Vocational Degree

☐ Bachelor's Degree

☐ Master's Degree

☐ Ph.D, J.D., or M.D.

OCCUPATION

1. What is your occupation?

____

2. Are you currently working?

☐ Full-time ☐ On leave

☐ Part-time ☐ Not working
