# Supplementary material for: Semantic priming supports infants’ ability to learn names of unseen objects
Source: PLoS One. 2021 Jan 7;16(1):e0244968. doi: 10.1371/journal.pone.0244968 (PMC7790528; doi:10.1371/journal.pone.0244968)
Supplement: S3 Appendix — *Source of images in the Test phase: https://unsplash.com. (PDF) [file pone.0244968.s003.pdf]

Appendix 3. Visual* and auditory stimuli in Experiments 1 and 2

Semantic Priming condition

Semantic neighborhood	Priming Phase					Test Phase
	Familiar word-object 1	Familiar word-object 2	Familiar word-object 3	Novel word		
Fruits: object images	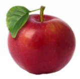	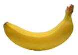	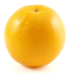			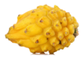 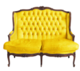
Fruits: auditory stream	Ooh! Look! An apple! Do you see the apple?	Ooh! Look! A banana! Do you see the banana?	Ooh! Look! An orange! Do you see the orange?	Ooh! A modi! That's a nice modi! I like modis!	Let's play a game! Let's find the modi!	Now look! Where is the modi? [2 s delay] Can you find the modi?
Vehicles: object images	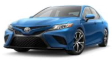	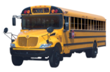	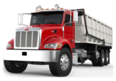			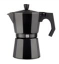 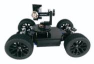
Vehicles: auditory stream	Ooh! Look! A car! Do you see the car?	Ooh! Look! A bus! Do you see the bus?	Ooh! Look! A truck! Do you see the truck?	Ooh! A dax! That's a nice dax! I like daxes!	Let's play a game! Let's find the dax!	Now look! Where is the dax? [2 s delay] Can you find the dax?
Animals: object images	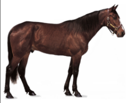	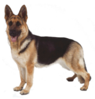	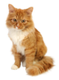			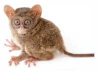 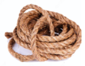
Animals: auditory stream	Ooh! Look! A horse! Do you see the horse?	Ooh! Look! A dog! Do you see the dog?	Ooh! Look! A cat! Do you see the cat?	Ooh! A wug! That's a nice wug! I like wugs!	Let's play a game! Let's find the wug!	Now look! Where is the wug? [2 s delay] Can you find the wug?
Clothing: object images	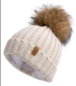	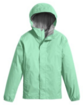	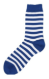			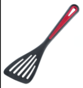 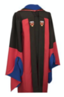
Clothing: auditory stream	Ooh! Look! A hat! Do you see the hat?	Ooh! Look! A jacket! Do you see the jacket?	Ooh! Look! A sock! Do you see the sock?	Ooh! A blicket! That's a nice blicket! I like blickets!	Let's play a game! Let's find the blicket!	Now look! Where is the blicket? [2 s delay] Can you find the blicket?

Follow-up condition

Semantic neighborhood	Priming Phase					Test Phase
	Familiar word-object 1	Familiar word-object 2	Familiar word-object 3	Novel word		
Fruits: object images	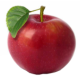	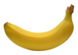	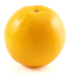			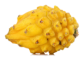 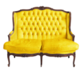
Fruits: auditory stream	Ooh! Look! An apple! Do you see the apple?	Ooh! Look! A banana! Do you see the banana?	Ooh! Look! An orange! Do you see the orange?	Ooh! A modi! That's a nice modi! I like modis!	Let's play a game! Let's find the modi!	Now look! Where is the *danu*? [2 s delay] Can you find the *danu*?
Vehicles: object images	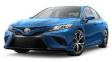	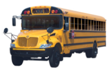	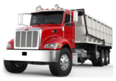			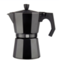 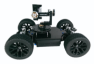
Vehicles: auditory stream	Ooh! Look! A car! Do you see the car?	Ooh! Look! A bus! Do you see the bus?	Ooh! Look! A truck! Do you see the truck?	Ooh! A dax! That's a nice dax! I like daxes!	Let's play a game! Let's find the dax!	Now look! Where is the *lif*? [2 s delay] Can you find the *lif*?
Animals: object images	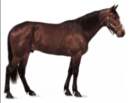	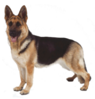	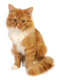			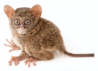 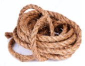
Animals: auditory stream	Ooh! Look! A horse! Do you see the horse?	Ooh! Look! A dog! Do you see the dog?	Ooh! Look! A cat! Do you see the cat?	Ooh! A wug! That's a nice wug! I like wugs!	Let's play a game! Let's find the wug!	Now look! Where is the *neem*? [2 s delay] Can you find the *neem*?
Clothing: object images	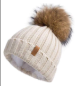	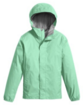	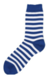			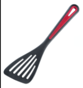 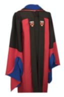
Clothing: auditory stream	Ooh! Look! A hat! Do you see the hat?	Ooh! Look! A jacket! Do you see the jacket?	Ooh! Look! A sock! Do you see the sock?	Ooh! A blicket! That's a nice blicket! I like blickets!	Let's play a game! Let's find the blicket!	Now look! Where is the *toma*? [2 s delay] Can you find the *toma*?

No Priming condition

Semantic neighborhood	Priming Phase					Test Phase
	Familiar word-object 1	Familiar word-object 2	Familiar word-object 3	Novel word		
Fruits: object images	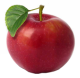	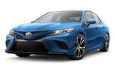	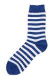			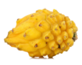 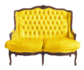
Fruits: auditory stream	Ooh! Look! An apple! Do you see the apple?	Ooh! Look! A car! Do you see the car?	Ooh! Look! A sock! Do you see the sock?	Ooh! A modi! That's a nice modi! I like modis!	Let's play a game! Let's find the modi!	Now look! Where is the modi? [2 s delay] Can you find the modi?
Vehicles: object images	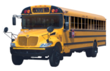	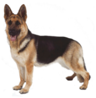	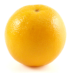			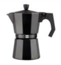 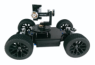
Vehicles: auditory stream	Ooh! Look! A bus! Do you see the bus?	Ooh! Look! A dog! Do you see the dog?	Ooh! Look! An orange! Do you see the orange?	Ooh! A dax! That's a nice dax! I like daxes!	Let's play a game! Let's find the dax!	Now look! Where is the dax? [2 s delay] Can you find the dax?
Animals: object images	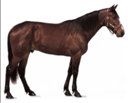	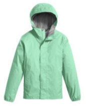	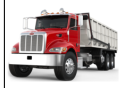			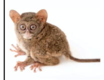 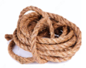
Animals: auditory stream	Ooh! Look! A horse! Do you see the horse?	Ooh! Look! A jacket! Do you see the jacket?	Ooh! Look! A truck! Do you see the truck?	Ooh! A wug! That's a nice wug! I like wugs!	Let's play a game! Let's find the wug!	Now look! Where is the wug? [2 s delay] Can you find the wug?
Clothing: object images	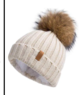	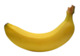	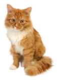			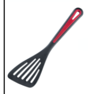 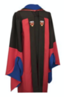
Clothing: auditory stream	Ooh! Look! A hat! Do you see the hat?	Ooh! Look! A banana! Do you see the banana?	Ooh! Look! A cat! Do you see the cat?	Ooh! A blicket! That's a nice blicket! I like blickets!	Let's play a game! Let's find the blicket!	Now look! Where is the blicket? [2 s delay] Can you find the blicket?

*Source of images in the Test phase: https://unsplash.com
