# Supplementary material for: Semantic priming supports infants’ ability to learn names of unseen objects
Source: PLoS One. 2021 Jan 7;16(1):e0244968. doi: 10.1371/journal.pone.0244968 (PMC7790528; doi:10.1371/journal.pone.0244968)
Supplement: S4 Appendix — (PDF) [file pone.0244968.s004.pdf]

1. Did you complete the book reading session today? Yes/No
2. How many words did you complete in today's session? If you did not complete the entire book today, please indicate the reason (e.g., the child became disengaged, cried, etc.). Please type your response in the box below.

| |
|---|
| |

3. How engaged was your child during today's reading session?
   a. Did he/she look at the pictures?

      Yes No
   b. Did he/she point?

      Yes No
   c. Did he/she vocalize?

      Yes. No
4. Please provide any additional comments in the box below (e.g., does your child enjoy the reading sessions? Do you have an impression that he/she has any preferences for specific images and words, etc.)

| |
|---|
| |
