# Supplementary material for: Semantic priming supports infants’ ability to learn names of unseen objects
Source: PLoS One. 2021 Jan 7;16(1):e0244968. doi: 10.1371/journal.pone.0244968 (PMC7790528; doi:10.1371/journal.pone.0244968)
Supplement: Attachment — Submitted filename: Response to Reviewers.docx [file pone.0244968.s005.docx]

Dear Dr. Butler,

Please consider our revised manuscript PONE-D-20-14424 for publication in PLOS ONE. We are grateful to you and the reviewers for your thoughtful feedback. We hope that you agree that by responding to this very productive set of reviews, we have produced a significantly stronger manuscript. Please see below point-by-point responses to reviewer’s comments.

Sincerely,

Elena Luchkina

Summary of the major changes:

1. We clarified that the central construct under investigation is “abstract verbal reference”, which we define operationally as “an appreciation that words are linked to mental representations that can be established, retrieved and modified, even when the entities to which they refer are not perceptually available”. See §1 of the Introduction, as well as §2 about “displaced/fragmented reference” as its operationalized forms.
2. Following R1’s and R’s excellent suggestion, we no longer frame the work in the literature on superordinate category knowledge. We now focus on semantic neighborhoods as our main experimental manipulation. We have changed the title to reflect this modification and renamed the conditions from Category Priming and No-Category Priming conditions to Semantic Priming and No Priming conditions respectively.
3. Based on R1’s suggestion, we redesigned the Follow-up Control condition. It now addresses the possibility that success in the Semantic Priming condition is due to semantic relations among the objects themselves and is not dependent upon infants’ inferences about novel word meanings.
4. Per R2’s suggestion, we expanded the introduction to better situate the current project within the word-learning and reference literatures.
5. We also modified the test stimuli included in all experiments and conditions to address R2’s suggestion to minimize perceptual distance between target and distracter images.

Reviewer 1

*The reviewer suggested that the test for our hypothesis would be stronger if we do not to locate our predictions in relation to superordinate category knowledge in infants this young.*

We are grateful to R1 for the suggestion. We agree that we need not rely upon superordinate category knowledge to test abstract verbal reference. Instead, we frame the hypothesis in the context of the semantic priming available from known words. We propose that the ability to establish a representation of a hidden referent based on a semantic neighborhood would constitute strong evidence of resolving abstract reference (see p. 6, §1).

*The reviewer also raised a concern that infants’ looking behavior in the Semantic Priming condition (formerly “Category Priming condition”) could be driven by the relations between the objects rather than by infants’ inferences about the meaning of the novel word.*

We thank the reviewer for bringing this up. In response, we changed the design of the Follow-up Control condition, which now addresses this concern. Now, the Follow-up Control condition is designed identically to the Semantic Priming condition, except that infants hear an unfamiliar novel noun during test trials (see p. 10, §1). This way, we can discern between the effects of the relation between the objects and infants’ semantic inferences about the novel word.

*The reviewer asked that we provide power analysis for our sample, taking into account the drop-out rate, and indicate what multiple corrections would be applied.*

We now added a power analysis and a recruitment plan that incorporates the projected drop-out rate (p. 6, §2). Because our planned analyses are based on GLMM, which (as we now point out) includes Test Item as a random factor, we do not need additional multiple corrections.

*The reviewer suggested that we use a completely novel word during Test in the Follow-up Control condition to evaluate whether infants associated the word with the target object.*

As mentioned above, we have done so. (p. 12, §1).

Reviewer 2

*The reviewer asked that we ground our work more comprehensively in the literature on word learning and outline the new insights that our work will bring to the problem of verbal reference.*

We appreciate this suggestion and have done so, now including the references cited by the reviewer and many others (pp. 3-4). We highlighted the difference between the extant empirical investigations and the current proposal, as well as our contribution to this literature (p. 4, §2; *“What remains unknown is when infants successfully acquire new representations and update them on the basis of language alone.”*).

*The reviewer also suggested we define more clearly the construct under investigation.*

We appreciate this suggestion. We clarified that the central construct under investigation is “abstract verbal reference”, which we define on p. 1, §1.

*The reviewer asked that we explicitly state our predictions for each condition.*

We have now added explicit predictions for each condition (pp. 12-13).

*R2 suggested that we minimize the perceptual differences between test images within and across conditions.*

We appreciate this suggestion, and have edited the test stimuli accordingly (see Appendix 3).

*The reviewer asked us to clarify the advantages of the Follow-up Control condition, pointing out how they help address the research question.*

We appreciate this suggestion. In response, we have clarified that the Follow-up Control condition (which now has been modified following R1’s suggestion; see p. 10, §1) will help discern between the effects of the relation between the objects and infants’ semantic inferences about the novel word.

*The reviewer suggested that we clarify how we decided on the duration of test trials and that we outline exclusion criteria for the looking data.*

We now articulate the reasons for 6 s test trials and the threshold criteria in the pilot and proposed studies. Both are based on earlier investigations of infants’ ability to take advantage of existing word knowledge to infer the meaning of novel words (p. 10, § 2; ).

*The reviewer asked that we explain the sample size and provide power analysis.*

We now added power analysis on p. 6, §2.

*The reviewer asked that we clarify why we chose to a within-subjects design with 4 test trials.*

We have done so on p. 8, §2.*“We selected a between-subject design to avoid potential carry-over effects, which would dilute the predicted advantage of Semantic Priming”*.

*The reviewer also asked us to explain to we would analyze the information collected from the demographic questionnaire and whether it would be linked to the DVs.*

The demographic data will used in preliminary analyses and may be included in the main analyses if the preliminary analyses show a significant effect. We do not have specific hypotheses about the role of the demographic factors in the development of abstract verbal reference comprehension. We clarify this on p. 12, §2.

*The reviewer suggested that we provide a COVID-19 contingency plan and outline an adjusted timeline for data collection.*

We added a COVID-19 contingency plan and explained the projected timeline of data collection on p. 7, §2.
